# “We're on the ground, we know what needs to be done”: Exploring the role of Aboriginal Health Workers in primary health care

**DOI:** 10.3389/fpubh.2022.1010301

**Published:** 2023-01-19

**Authors:** Ragavi Jeyakumar, Bindu Patel, Julieann Coombes, Ty Madden, Rohina Joshi

**Affiliations:** ^1^The George Institute for Global Health, University of New South Wales, Sydney, NSW, Australia; ^2^Faculty of Medicine and Health, University of New South Wales, Sydney, NSW, Australia; ^3^School of Population Health, University of New South Wales, Sydney, NSW, Australia; ^4^The George Institute for Global Health, New Delhi, India

**Keywords:** Aboriginal Health Worker, community health worker, primary health care, workforce, sustainability

## Abstract

**Background:**

Aboriginal Health Workers (AHWs) are core providers of primary health care (PHC) for First Nations peoples in Australia. However, the national AHW workforce is aging and in short supply. There is a poor understanding of the factors contributing to this attrition from the perspectives of AHWs themselves. This study aimed to systematically explore the current functioning and sustainability of AHWs in NSW PHC by amplifying AHW voices.

**Materials and methods:**

This study was co-designed with three Aboriginal health services in NSW. It included a literature review exploring the role of AHWs in NSW, and yarns with AHWs and their supervisors at participating services. Yarning is an Indigenous approach to knowledge generation centered upon storytelling. The yarns were guided by the USAID-developed Community Health Worker Assessment and Improvement Matrix. Yarn transcripts were analyzed using cyclical thematic analysis to identify key facilitators and challenges for AHW practice.

**Results:**

The yarns highlighted five categories of change that are required to ensure AHW sustainability: community connection, recognition, value, support, and an inclusive health system. The yarns revealed that there are both service- and system-level factors influencing each of these categories of change.

**Conclusions:**

The lived experiences of AHWs in NSW emphasize five key categories of change that are required to ensure workforce sustainability. It is evident that a system-wide paradigm shift to better include holistic approaches to health is necessary to truly ensure sustainability. Co-designing similar studies with ACCHOs across NSW can help inform this change.

## 1. Introduction

Since the establishment of the first Aboriginal Community Controlled Health Organisation (ACCHO) in 1971, Aboriginal Health Workers (AHWs) have played a significant role in the provision of primary health care (PHC) for Aboriginal and Torres Strait Islander peoples (1). Australia's diverse First Nations peoples hold a holistic understanding of health, encompassing health of the body, mind, spirit, and land that is best served by Aboriginal community controlled organisations (2). While mainstream PHC in Australia is typically delivered by general practice clinics, PHC for First Nations populations within Australia is driven by 196 Aboriginal PHC services operating nationally. Aboriginal PHC services include both 144 ACCHOs and 52 state-run Aboriginal Medical Services (3). The services seek to provide whole-of-community, culturally safe, accessible PHC for First Nations peoples (4). AHWs are based within these services and play a central role in achieving these aims (5).

AHWs are health workers that are members of the First Nations communities where they work that have been trained to support holistic PHC provision for their communities (6). The AHW role is one of few positions within the Australian health workforce that is exclusively occupied by First Nations peoples (5). As such, they are instrumental in creating a culturally safe and responsive health system (2, 5). As members of the communities in which they practice, AHWs have an innate understanding of the strengths, concerns and lived experiences of the people they serve, and this uniquely positions them as “cultural brokers” between the community and the health system (6). Due to the community-specific nature of their practice, there is great variability in the roles performed by AHWs based on community needs, with responsibilities ranging from clinical task-sharing to community education (7–9). Studies have shown that AHWs improve the uptake of preventive services, screening programs and chronic disease treatment in their communities by facilitating culturally appropriate care, reducing communication gaps, and enhancing referral linkages (10–14). Additionally, beyond improving measurable outcomes, AHWs are able to create cultural change within mainstream health services. They have been seen to act as patient advocates and clinician educators to reconcile the Western biomedical model of healthcare with First Nations understandings of health (9, 15). Government health strategies increasingly position AHWs as central to “Closing the Gap” and place high expectations on the workforce's capacity to achieve universal health access for First Nations peoples (5, 16–18). Indeed, the Australian Government's “Primary Health Care 10 Year Plan” aims to have a “continually growing” Aboriginal health workforce in 10 years “to support the health needs of Aboriginal and Torres Strait Islander peoples”(18).

**Figure 1:**
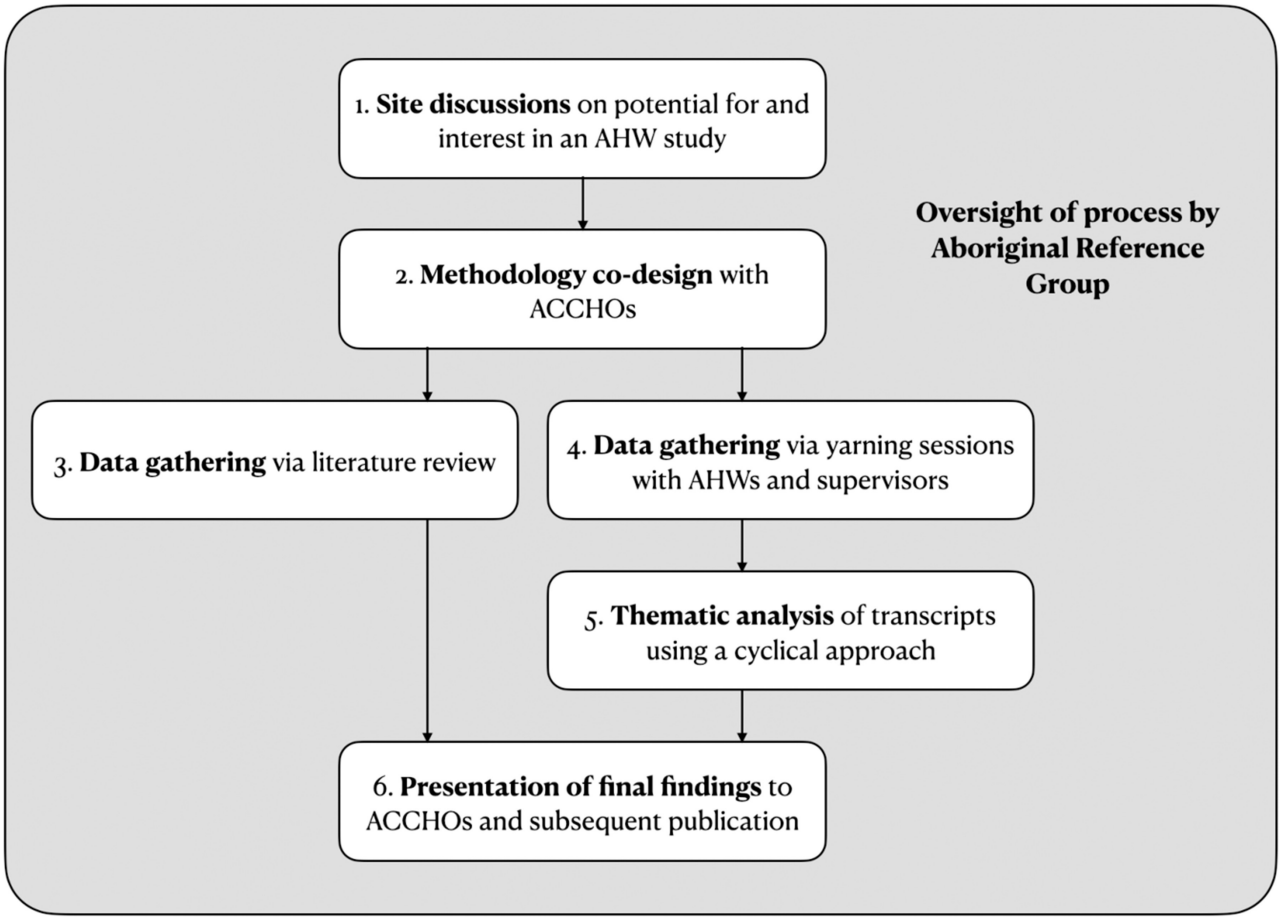
Study design. This flowchart shows the steps involved in “Exploring the Role of Aboriginal Health Workers in PHC in New South Wales”. AHW, Aboriginal Health Worker; ACCHO, Aboriginal Community Controlled Health Organisation.

As of 2020, there were 842 AHWs working across the 196 Aboriginal PHC services nationally (3). Over 25% of these AHWs were based in Aboriginal health services in New South Wales (NSW), where AHWs work as members of an integrated healthcare team to provide “flexible, holistic and culturally sensitive health services” to Aboriginal communities (3, 19). There are four broad categories of AHW recognized in NSW: Aboriginal Health Worker, Aboriginal Health Practitioner, Senior Aboriginal Health Worker, and Principal Aboriginal Health Worker (20). There are many specific AHW roles within each of these categories, and their responsibilities and training requirements are prescribed by the NSW Health Aboriginal Health Worker Guidelines (20). The typical position of AHWs within an Aboriginal PHC service is summarized in Figure 1. This structure was synthesized from a number of sources (3, 4, 19–24) and confirmed through our research work. It should be noted that due to the community-dependent nature of ACCHOs, there is no fixed state-wide structure for AHWs within ACCHOs and variation is expected (24).

It is increasingly clear that the sustainability of AHWs in the NSW and Australian workforce is threatened, with studies reporting of low self-worth and high levels of stress and attrition amongst AHWs (6, 10). The national AHW workforce is stagnant and aging, with the number of AHWs falling from 221 to 207 staff per 100,000 First Nations people between 2006 and 2016, and the proportion of AHWs aged 55–64 rising by 7.5% in the same period (10). This presents a significant threat to the ongoing safety of PHC for First Nations peoples. Several factors have been speculated to contribute to this phenomenon. Most significantly, it has been noted that there remains a pervasive lack of understanding of the unique skills, roles, and value of AHWs (1, 6, 25–28). In daily operations, this has been reported to manifest as a lack of respect for the AHW role amongst managers and other health professionals (26, 27) and poor role boundaries in the workplace (9) leading to the frequent relegation of AHWs to “menial” administration and transport tasks (28, 29). At an institutional level, it has been identified that the lack of value for the role has impeded the inclusion of AHWs in formal workforce planning, led to lack of competitive pay, and prevented the establishment of adequate, sustainable funding structures (7, 8). For instance, at present, AHW salaries in ACCHOs are not regulated statewide and are determined through enterprise bargaining processes with individual ACCHOs (9).

Few studies have sought to holistically explore the day-to-day functioning, strengths, and limitations of the AHW workforce. Most literature on AHWs in Australia has focussed on evaluating the role of AHWs in one-off, novel interventions (12, 14, 28–30), rather than within the PHC system itself. Indeed, no paper or organizational policy clearly describes the position of AHWs within PHC. Any studies which have investigated the typical functioning of the AHW program have tended to focus on isolated components, such as defining the role's responsibilities (8) or investigating its support structures (29). Additionally, AHW voices have been notably absent from almost all investigations (9). A comprehensive understanding of the position, strengths, and limitations of the current AHW PHC program, from the perspectives of the AHWs that work within it, is essential to ensuring the role's sustainability. This knowledge has the potential to strengthen the AHW program by identifying its key supports and challenges. Studies of AHWs that are founded upon decolonised research methodologies and Indigenous approaches to data gathering could effectively gather such knowledge (31). No such study has been conducted in the state of NSW, which contains 37 of the 143 ACCHOs currently operating in Australia (32). As of 2020, there were 220 AHWs working in Indigenous-specific PHC across NSW (3).

There is growing interest in the use of community health worker (CHW) programs to address health inequity in high-income countries internationally (33, 34). For instance, the Australian Government has recently committed to trialing a “Rural Area Community Controlled Health Organisation” model of health delivery in remote areas, potentially involving rural CHWs, and have increased use of CHWs for refugee populations (18). Understanding the factors which contribute to a sustainable AHW workforce will strengthen the existing AHW program. Additionally, it could help guide the expansion and implementation of other CHW programs. Therefore, the overall aim of this study was to work with AHWs to uncover their knowledge in a culturally safe manner and to enable their lived experiences to guide the development of a safer, fairer health system for all Australians. The study sought to use Indigenous research methodologies, such as yarning, to achieve this aim.

## 2. Materials and methods

We used the Community Health Worker Assessment and Improvement Matrix (CHWAIM) which is an internationally recognized tool that has been developed to guide the systematic analysis of CHW programs (Table 1) (35). The CHWAIM examines ten components of a CHW program that are evidenced to be essential to their functioning (35). By exploring each of these components, the CHWAIM can comprehensively uncover the strengths and limitations of any CHW program and systematize understanding of its present workings.

**Table 1:** CHWAIM assessment framework (35).

Role and recruitment	How the community, CHW, and health system design and achieve clarity on the CHW role and from where the CHW is identified and selected
**Training**	How pre-service training is provided to the CHW to prepare for his/her role and ensure s/he has the necessary skills to provide safe and quality care; and, how ongoing training is provided to reinforce initial training, teach CHWs new skills, and to help ensure quality
**Accreditation**	How health knowledge and competencies are assessed and certified prior to practicing and recertified at regular intervals while practicing
**Equipment and supplies**	How the requisite equipment and supplies are made available when needed to deliver expected services
**Supervision**	How supportive supervision is carried out such that regular skill development, problem-solving, performance review, and data auditing are provided
**Incentives**	How a balanced incentive package reflecting job expectations, including financial compensation in the form of a salary, and non-financial incentives, is provided.
**Community involvement**	How a community supports the creation and maintenance of the CHW program.
**Opportunity for advancement**	How CHWs are provided career pathways.
**Data**	How community-level data flow to the health system and back to the community and how they are used for quality improvement
**Linkages to the national health system**	The extent to which the Ministry of Health has policies in place that integrate and include CHWs in health system planning and budgeting and provide logistical support to sustain district, regional and/or national CHW programs.

This table outlines the Community Health Worker Assessment and Improvement Matrix (CHWAIM), developed conjointly by the World Health Organisation and the United States Agency for International Development Healthcare Improvement Project in 2011. The CHWAIM outlines 10 components evidenced to contribute to highly functional CHW programs. It was developed as part of a toolkit that also includes questionnaires, worksheets, and recruitment forms to aid in the assessment and improvement of CHW programs (35).

CHW, Community Health Worker.

This study aims to apply the CHWAIM to systematically explore the functioning and sustainability of AHWs in NSW PHC, by amplifying AHW voices. We were interested to understand the current role of AHWs in PHC in NSW and the day-to-day and systematic factors that impact the AHW program sustainability.

### 2.1. Setting

This study involved three health services operated by two ACCHOs in New South Wales (NSW). All three sites were in regional NSW and collectively employed 21 AHWs. NSW was selected as a setting for the study due to lack of existing AHW research within the state and existing research relationships. Over 25% of practicing AHWs in Australia are based in NSW and the governance of AHWs is similar to other states (3).

### 2.2. Study design

**Figure 2:**
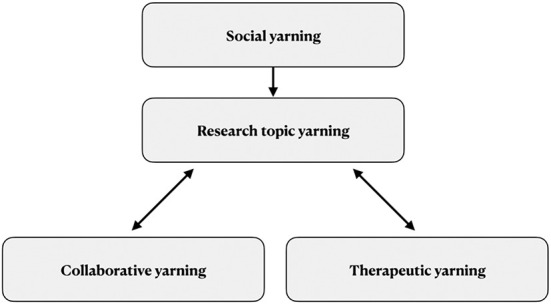
The yarning research process (36). This flowchart created by Ng'andu and Bessarab outlines the four different types of yarns that are involved in research through yarning (36). Firstly, there is social yarning: an unstructured conversation that takes place before the research occurs to build trust and accountability by identifying yourself and your experiences. Subsequently, there is research topic yarning to collect information pertaining to the research question through the participants' stories. The research topic yarn can transition to collaborative yarning, in which information about the research project and ideas are shared, and to therapeutic yarning, which takes place when a participant discloses traumatic or sensitive information. The researcher transitions to the role of a listener, to help affirm and facilitate the participant's meaning-making in voicing their story.

This study was co-designed with the participating ACCHOs in a series of phases as summarized in Figure 2. The study's research team consisted of Aboriginal (JC, TM) and non-Indigenous (RaJ, RoJ, BP) researchers. The team was guided by an Aboriginal Reference Group composed of First Nations researchers and community members with diverse experiences within the health sector.

Our study aimed to understand the current role of AHWs in PHC in NSW and the day-to-day and factors that impact AHW program sustainability through the voices of AHWs. These aims were met through yarning at the participating health services.

#### 2.2.1. Site discussions

In the initial research phase, JC contacted two ACCHOs and TM visited 15 ACCHOs across NSW to discuss their interest in this study. We sought feedback regarding the plan's value, viability, and appropriateness. This feedback was used to finalize the study aims and design. While numerous ACCHOs contributed to the pre-study consultation process, data was only collected at three sites due to time constraints.

#### 2.2.2. Methodology co-design

A central aim of this study was to amplify AHW voices. The research team and participating communities identified that to achieve this, our work had to be founded on culturally safe methods of knowledge creation. Yarning, a validated Indigenous research methodology (31, 36–40), was consequently selected as our data collection method. Yarning is an approach to qualitative data gathering that centers storytelling in conversation (36). Unlike questionnaires or surveys which are fixed according to the researchers' agenda, yarns are guided by the stories and lived experiences of participants. Storytelling is a key component of First Nations pedagogy and its use in yarning allows research to be conducted in a manner that assumes responsibility, reciprocity, and respect for Aboriginal Ways of Knowing, Being, and Doing (31, 40, 41).

**Figure 3:**
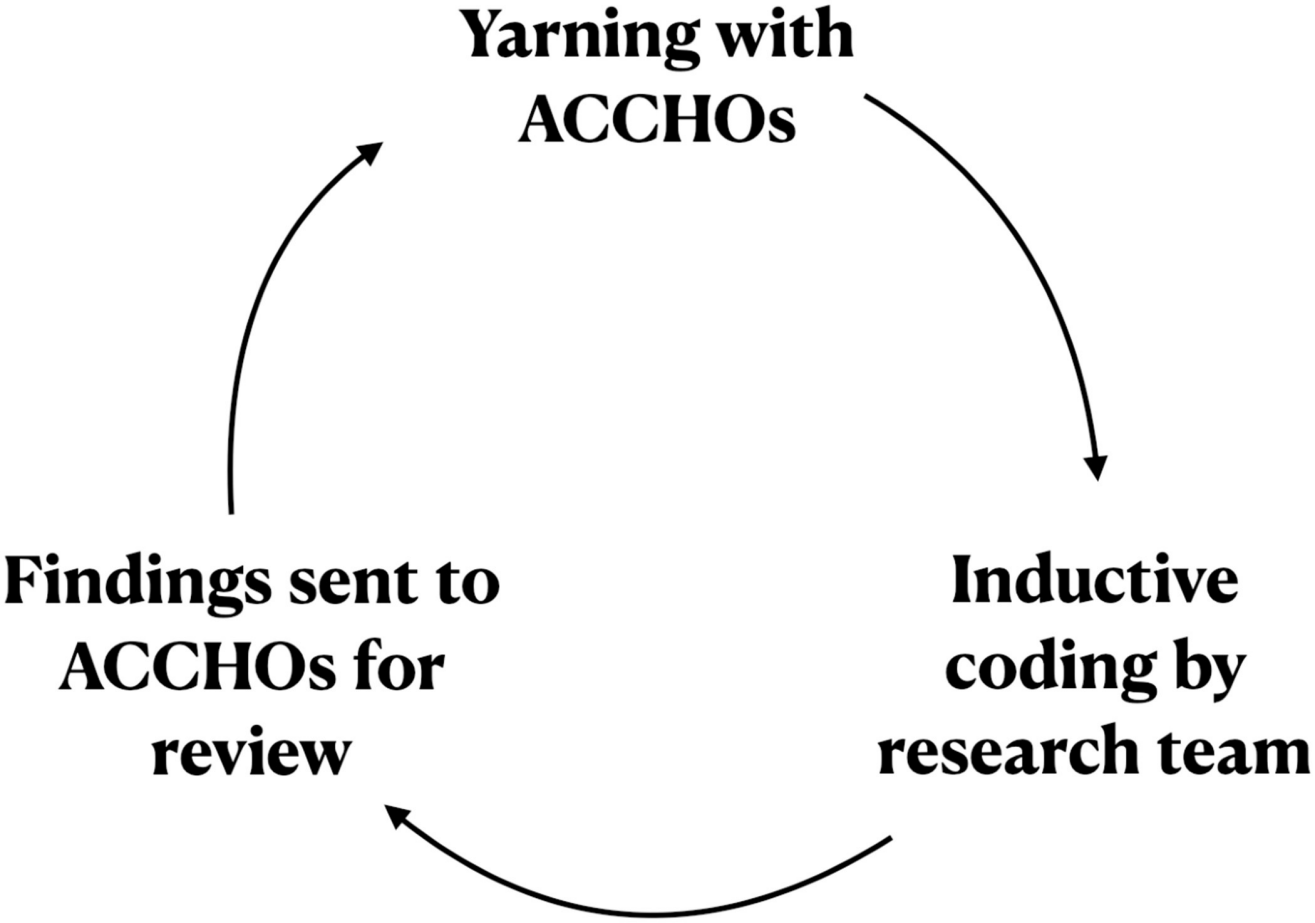
A cyclical approach to thematic analysis. This flowchart summarizes the cyclical approach to analysis utilized in this study. ACCHO, Aboriginal Community Controlled Health Organisation.

The yarning process in this study sought to gather stories from AHWs about their lived experience in the field and stories from AHW supervisors about the governance of AHWs in NSW. Ng'andu and Bessarab outline that yarning sessions in research progress through four different types of yarns: social, research topic, collaborative and therapeutic (Figure 3) (36). Firstly, there is social yarning: an unstructured conversation that takes place before the research occurs to build trust and accountability by each participant and researcher identifying themselves and their experiences. Subsequently, there is research topic yarning to collect information pertaining to the research question through the participants' stories. The research topic yarn can transition to collaborative yarning in which information about the research project and ideas are shared, and to therapeutic yarning which takes place when a participant discloses traumatic or sensitive information. Throughout the process, the researcher transitions primarily to the role of a listener, to help affirm and facilitate the participant's meaning-making in voicing their story.

A yarn guide was developed in conjunction with the ACCHOs to frame the research topic yarns in this study. The guide (Supplementary material) was based on the CHWAIM to help ensure that the key aspects of the AHW program were addressed in each yarn.

#### 2.2.3. Yarning

Yarns were conducted with two groups of staff at each ACCHO: AHWs, and AHW supervisors. Group yarns were conducted with all participating AHWs at each site. Separate supervisor yarns were conducted with either individuals or pairs of supervisors. A total of 13 AHWs (62% of all employed AHWs) and five supervisors participated in the study, and a total of seven yarns were conducted.

The ACCHOs which consented to participate in the study recruited participants *via* email. Administrative staff shared email invites with all AHWs at each site and interested AHWs returned written consent. The yarns involved AHWs representing different roles and career stages at each ACHHO. Relevant supervisors were invited by the CEO of each ACCHO. All nominated supervisors participated in the study.

Due to travel restrictions imposed by the COVID-19 pandemic, in-person data collection was not possible. Consequently, all yarn sessions were held in a hybrid model, with researchers and an Aboriginal research associate (TM) facilitating remotely *via* Zoom, and ACCHO staff meeting in person. All yarns were audio-recorded with permission. The yarns lasted between 50 and 91 min and explored the topics outlined in the CHWAIM yarn guide (Supplementary material). Audio recordings of the yarns were deidentified and transcribed verbatim.

#### 2.2.4. Data analysis

**Figure 4:**
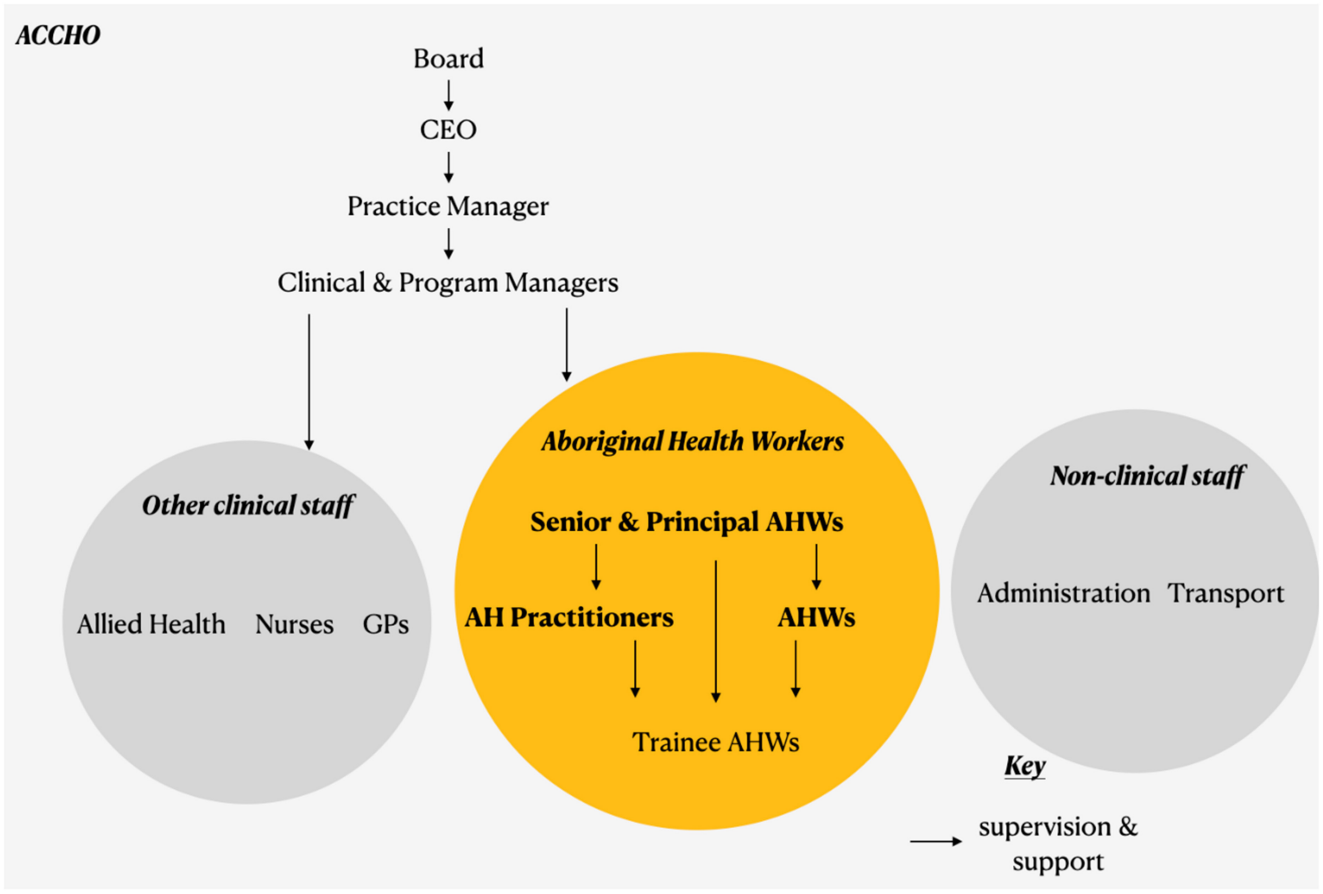
Position of AHWs in PHC in NSW. This chart summarizes the typical position and governance of AHWs (in the orange circle) within a typical NSW ACCHO. Other health staff are shown in the gray circles. The arrows signify the direction of supervision, starting with the Board. AHW, Aboriginal Health Worker; ACCHO, Aboriginal Community Controlled Health Organisation.

The yarn transcripts were explored using a cyclical approach to thematic analysis (Figure 4). This approach firstly involved Aboriginal (JC, TM) and non-Indigenous (RaJ, RoJ, BP) researchers examining the transcripts together. Data was analyzed using a mixed approach, which involved deductive and inductive identification of key themes iteratively over several rounds and categorized into codes and sub-codes using NVivo V.12 (QSR International, 2015). Secondly, the preliminary themes emerging from this process were summarized and sent back to the ACCHOs for review. A discussion session was then organized *via* Zoom to facilitate collaborative yarning between ACCHOs and the research team. Following this discussion, themes and codes were again refined by the research team. This cyclical approach presented an analytical parallel of yarning methodology in generating findings that were grounded in Aboriginal Ways of Knowing, Being, and Doin g(42).

#### 2.2.5. Presentation of findings

The key findings from the study were summarized in a plain-language results paper and presented back to each site in an online workshop for participant feedback.

### 2.3. Ethics approval

This project received approval from the UNSW Human Research Ethics Committee (reference: HC210247) and the Aboriginal Health and Medical Research Council Human Research Ethics Committee (reference: 1800/21).

## 3. Results

### 3.1. Facilitators and challenges for sustainability

The yarns highlighted several core facilitators and challenges for AHW sustainability in NSW. These facilitators and challenges can be mapped to seven of the 10 components of the CHWAIM, as summarized in Table 2.

**Table 2:** Facilitators and challenges for AHW sustainability at participating sites.

	**Facilitators**	**Challenges**
Role and recruitment	• Adaptability of role to community and ACCHO needs • Internal recruitment to upskill and recognize community	• Fragmentation of work • Limited decision-making and clinical capacity • External recruitment dependent on community perception
Training	• Financial support for mandatory and additional training	• Ensuring holistic support for training • Lack of formalized, sustainably funded training pathways
Supervision	• Informal cultural mentorship by senior AHWs	• Embedding pathways for regular communication with official management • Embedding wellbeing support
Incentives	• Regular enterprise bargaining processes	• Lack of commensurate, competitive pay • Lack of adequate detail in award
Community involvement	• Strong AHW-community connection • Consultations to maintain ACCHO-community connection	• Constant AHW accountability to community
Opportunity for advancement	• Internal pathways for AHW advancement	• Lack of formalized advancement pathways • Fear of community disconnection with advancement
Linkages to the national health system	• Brokerage between community and national health system	• Lack of value for AHWs in Western system • Lack of value for ACCHOs in Western system

This table summarizes the key facilitators and challenges for AHW sustainability that were identified in our yarns. These key factors align with seven of the 10 components of the CHWAIM. The remaining three components (Accreditation, Equipment and supplies, and Data) were not prominent in the gathered stories of the AHWs.

AHW, Aboriginal Health Worker; ACCHO, Aboriginal Community Controlled Health Organisation

#### 3.1.1. Role and recruitment

AHWs at each site described their roles as broad and flexible, encompassing clinical, administrative, and client advocacy duties. Every AHW emphasized that their primary motivation was to support their community. The flexibility inherent in the AHW role allowed them to adapt to best serve the needs of the community and the community-controlled service in which they worked.

“*We have the general responsibilities—advocacy, supporting mob, accessing services, and coordinating clinics is probably a few of our main responsibilities here. But we have become so flexible that we're responsible in all aspects of the daily running of the clinic, whether that's jumping up, supporting transport, going to pick up a client to sitting with them within a consult and advocating for them in that part… so that we can still maintain a professional flow of service for our community because we know if one falls down, generally we all fall down.”*

However, AHWs described that the variability of their role meant that day-to-day work was often highly fragmented. This led to separation within AHW teams and contributed to burnout.

“*I'm the only one that does the Brokerage in CCS, so I'm burnt out 200% all the time. Like, yeah, so it's hard.”*

Further, a major concern voiced in every yarn was the limited capacity for decision-making and clinical practice within the AHW role. This limited scope failed to recognize the capabilities of AHWs. Supervisors highlighted that expanding this scope could help fill service gaps.

“*We're on the ground, we know what needs to be done.”*

“*They're trained to do much more than what they're actually doing… We probably could utilize our Aboriginal health workers in much more efficacy across the service if the scope of practice was broadened for the health workers. For example, the nurses that are currently doing vaccinations [are] being absolutely smashed. But I've got two nurses currently on that could have been opportunities for Aboriginal health workers to be supporting them in that process.”*

The yarns revealed that recruitment at each site centered on internal processes. Recruitment for entry-level AHW positions was typically from other areas of the ACCHO such as reception or transport. Management positions were also often advertised internally at first. This approach was recognized by participants as a clear way to demonstrate value for AHWs and their communities.

“*There's a policy here where we give people opportunities to bring people in as trainees, maybe at reception, and then they'll often move on to other roles within the organization … They're given, not preferential treatment, but they're notified about those vacancies and encouraged to apply. There are a lot of our staff who've been here for a long time, and they've worked their way through the organization.”*

Alternately, supervisors described that the viability of external recruitment was dependent on the reputation of the ACCHO within the community. A positive community perception was essential to ensuring that new AHWs could be attracted.

“*We're struggling to try and find people for roles, as well … if they've heard how people have been treated in the past or somebody has worked here and they haven't liked it and they've gone back out, Koori grapevine is faster than Facebook, I'll tell you.”*

#### 3.1.2. Training

AHWs received financial support from ACCHOs for both mandatory and additional training. They described that this support helped them feel valued by the organization.

“*They give us the opportunity to do a lot of training and we just take it on board and go from there with it. And there's that support from our service, our CEO and clinical service manager and those people that are involved and that's something that they look at, just empowering Aboriginal health workers … whatever we like, we'll just send through requests and if it's relevant they'll give us the funding to do that and it's really great.”*

However, both AHWs and supervisors identified that beyond funding, other forms of support, such as academic mentoring and paid leave were also required to successfully complete training. This support was less commonly provided.

“*We've got to give a lot of staff a hell of a lot of support to get through those courses… The majority of local people that we employ have limited education and no qualification, no skills. So they come in very raw and a lot of them have limited literacy and numeracy skills, and that places a high expectation on them to get skilled.”*

Further, access to additional training was typically provided on an individual basis, and AHWs highlighted that a clear, formalized rationale could aid in career planning. However, supervisors explained that the unsustainable funding of ACCHOs made it difficult to implement a consistent training budget.

#### 3.1.3. Supervision

Despite not being included in formal supervision structures, senior AHWS played a vital role in the professional and cultural mentorship of newer AHWs at each site. They helped facilitate a sense of “family” that was central to job satisfaction and cultural safety for AHWs in the workplace. However, AHWs described that there was poor communication between AHWs and official management teams. While several communication pathways had been planned at each site,–from formal meetings to informal team huddles–participants reported that these were often abandoned in favor of competing demands.

“*Facilitator: Do you guys feel like your voices are heard on an organizational level?*

*P1, P2, P3: No*.

*P4: Can you tell by the silence from us? Awkward silence… There's got to be more communication between the mob upstairs and us down here too as well.”*

Ultimately, AHWs described mechanisms to support workplace wellbeing and address concerns such as burnout and work-life balance were lacking.

“*It shouldn't be up to the individual to really try to identify what needs to be done for our wellbeing. You know we're already trying to find those solutions for our community, for their wellbeing, so you know the expectation from us would be having our managers, having our supervisors identify key areas of supports within wellbeing and implementing that.”*

#### 3.1.4. Incentives

AHW salary in ACCHOs is guided by a Commonwealth award. At each site, AHWs were able to enter “enterprise bargaining” processes to negotiate their pay above this award. However, despite these processes, every participant identified that a key factor impeding AHW sustainability was the lack of adequate pay. Firstly, AHW salary was not commensurate for the work that they perform in enabling clients to access all billable services and providing constant community support.

“*We're not really paid for what we do. We're paid on the contract that we sign but with working in community, we don't switch off at five o'clock … it's an ongoing role within the community and I think that needs to be, well not so much reflected because you can't really put a price on that kind of service outside of the work hours, but I think that needs to change.”*

“*Whilst they don't sign off on the [health assessment], the health worker will do most of the underlying work to generate that information, so without the health worker a doctor would be doing that, and we'd be paying them three times the amount, to get that same payment.”*

Secondly, AHW salary was not competitive with other roles available to AHWs outside of ACCHOs.

“*A lot of them, you know, have families, they have kids and that, they've got a mortgage, they've got a car and it's, they can't afford it on the pay they get, well … whatever pays best next … And less stress. Woolworths even sometimes.”*

This was exacerbated by the fact that the award does not account for the variations in pay grade between different AHW roles, most notably for Senior AHWs. Additionally, supervisors described that bargaining processes and unsustainable funding lead to a lack of consistency and transparency in AHW salaries between ACCHOs.

#### 3.1.5. Community involvement

Community connection was identified to be the core driver of AHW practice. Every yarn emphasized that AHWs were inextricably linked to their community and a sense of positive community impact was central to AHW job satisfaction.

“*We're here for our community. To provide the holistic care for our community.”*

“*It makes us feel good at the end of the day that we done something for them, you know. And that's the only recognition that I'd like to get–from my patients.”*

Regular formal and informal consultations helped maintain a close, positive connection between the ACCHO and the community. However, such strong community ownership of the AHW program led to AHWs being held accountable by the community outside of work hours. The subsequent lack of work-life boundaries was identified to be a major contributor to AHW burnout.

“*Being a health worker is not a 9:00 to 5:00 job. It's a 24-seven job so they have to have a real commitment to the job, but it impinges on their personal life … they are held accountable by their community, and it seems the more dedicated they are, and the more passionate they are, the more they are under the scrutiny and criticism of their community. That's very difficult.”*

#### 3.1.6. Opportunity for advancement

Each site was committed to the advancement of AHWs and their community through training and internal promotions. The typical pathway of promotion was from reception and other administrative roles to AHW, then to AHP, and in a limited number of cases, to management positions. However, these pathways were not formalized and the requirements for advancement were not explicated.

“*There is a pathway, but it's not an official pathway, it's not a structured pathway… initially we'll advertise internally before we look outside, but we won't actually say, because you've done so many years and you've got this particular qualification, that you must get this particular role.”*

Further, AHWs expressed disinterest in “progressing” to a management role due to the associated disconnection from the community, which is the core motivator for their work.

#### 3.1.7. Linkages to the national health system

Participants at each site recognized the importance of AHWs in linking clients to other health services. Every AHW expressed great satisfaction at being able to broker the required care for their community. However, the yarns emphasized that the Western biomedical model of health which dominates healthcare in Australia is limited in its ability to support and value the work of AHWs and ACCHOs. Supervisors described that the focus on clinical practice and “mainstream” healthcare services has created an unsustainable, incomplete model of funding for ACCHOs and their AHWs. Every yarn highlighted the lack of value and importance for the work of AHWs amongst policymakers and other health professionals.

“*I think recognizing our sector, the community-controlled sector, as actually being the pre-eminent provider of Aboriginal health, and that the health workers are the key to that. I think having that recognition, people say those words sometimes, but there's very little demonstration of actually understanding what that means, and actually acting upon that. So, I think that would make a big difference. It would make people who work here feel that they are actually justified in choosing to come here, and actually valued in terms of what they do, truly valued.”*

“*We say we couldn't operate without health workers, but we end up talking about hard skills, about their scope of practice, and can they do immunisations, or can't they? But really, most of their value, from a community point of view, is not those things. We can find somebody that can put a needle in someone's arm, but we can't find somebody that actually understands the background of a client and knows why they've come into the clinic so upset, and can actually communicate with them correctly, and in the right way to actually get information they need, to move them forward, to support them. And I think that those “soft skills” shall we call them, are probably the most important things that they provide, but probably the things that we look at least, and value least.”*

## 4. Discussion

We conducted a literature review and subsequent yarns with AHWs and their supervisors at three Aboriginal PHC services to explore the current functioning of the AHW program in NSW. Our co-designed methodology was founded on First Nations research methods and satisfied all 14 criteria of the Aboriginal and Torres Strait Islander Quality Appraisal Tool developed by Harfield et al. (43). This enabled us to gain safe insights into the genuine lived experiences of AHWs and their supervisors. Our study is the first in NSW to directly gather the perspectives of AHWs regarding each key component of the AHW program, as defined by the CHWAIM. The CHWAIM has been validated as an assessment tool by international studies of CHW programs (33, 35). Applying it in our yarns helped establish a holistic understanding of AHW program functioning in NSW.

### 4.1. Changes for sustainability

**Figure 5:**
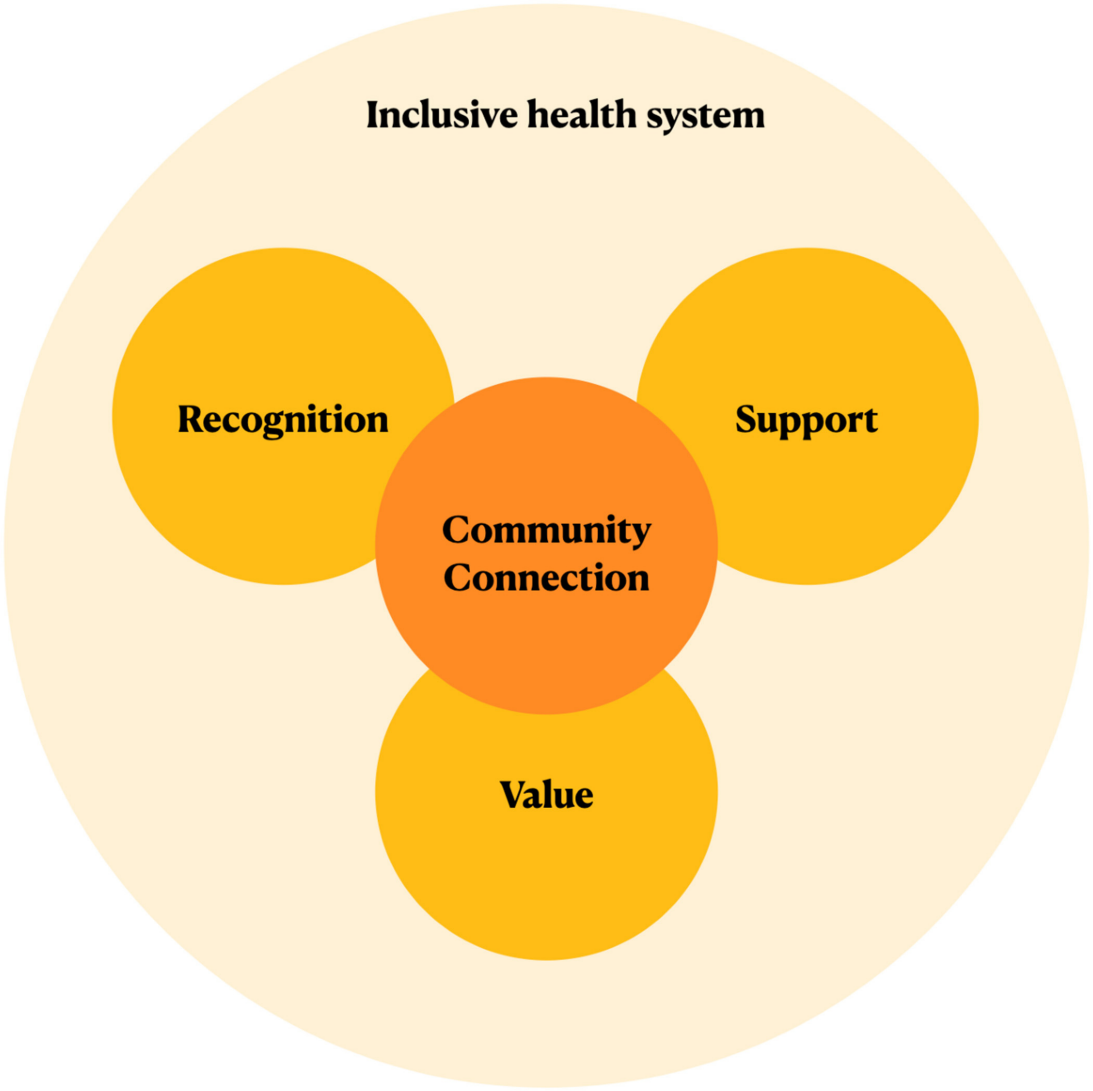
Five categories of change to ensure AHW sustainability. This figure summarizes the five categories of change for AHW sustainability emphasized by our yarns, with “Community Connection” central, and an ‘Inclusive health system' overarching.

The facilitators and challenges that arose from the yarns suggested five broad categories of change that are required in the creation of a sustainable workforce (Figure 5).

Community connection is central to AHW practice. The potential to create a positive community impact is vital both in attracting candidates to the AHW role and creating job satisfaction to increase AHW retention. The yarns suggested that an AHW role that is flexible enough to adapt to community needs and a positive ACCHO-community relationship are key to ensuring this connection. This connection should be prioritized even as AHWs advance within the organization.

Recognition of the importance and expertise of AHWs is vital in promoting their work. The education of stakeholders–including community members other health professionals–regarding the value of AHWs in the health system and the challenges of their work was identified to be essential in recognition. Further, the unique skills of AHWs should be acknowledged by expanding clinical practice and decision-making capacities to reflect their expertise.

Adequate workplace support is essential to prevent burnout amongst AHWs. Our yarns identified that such support should be holistic, encompassing academic, financial and wellbeing supports. Regular, open pathways for communication need to be prioritized and the importance of cultural supervision by senior AHWs should be recognized. Furthermore, value for the work of AHWs must be demonstrated through commensurate pay that is sufficiently competitive to ensure retention. Non-financial incentives such as opportunities for advancement and training opportunities should be sustainably funded and their requirements clearly articulated so that they are accessible to all AHWs.

All these changes need to be made within a healthcare system that is inclusive of holistic approaches to healthcare such as First Nations understandings of health. Policy changes are required to shift focus from billable clinical services to enable sustainable, complete funding for holistic care providers such as ACCHOs and their AHWs.

**Figure 6:**
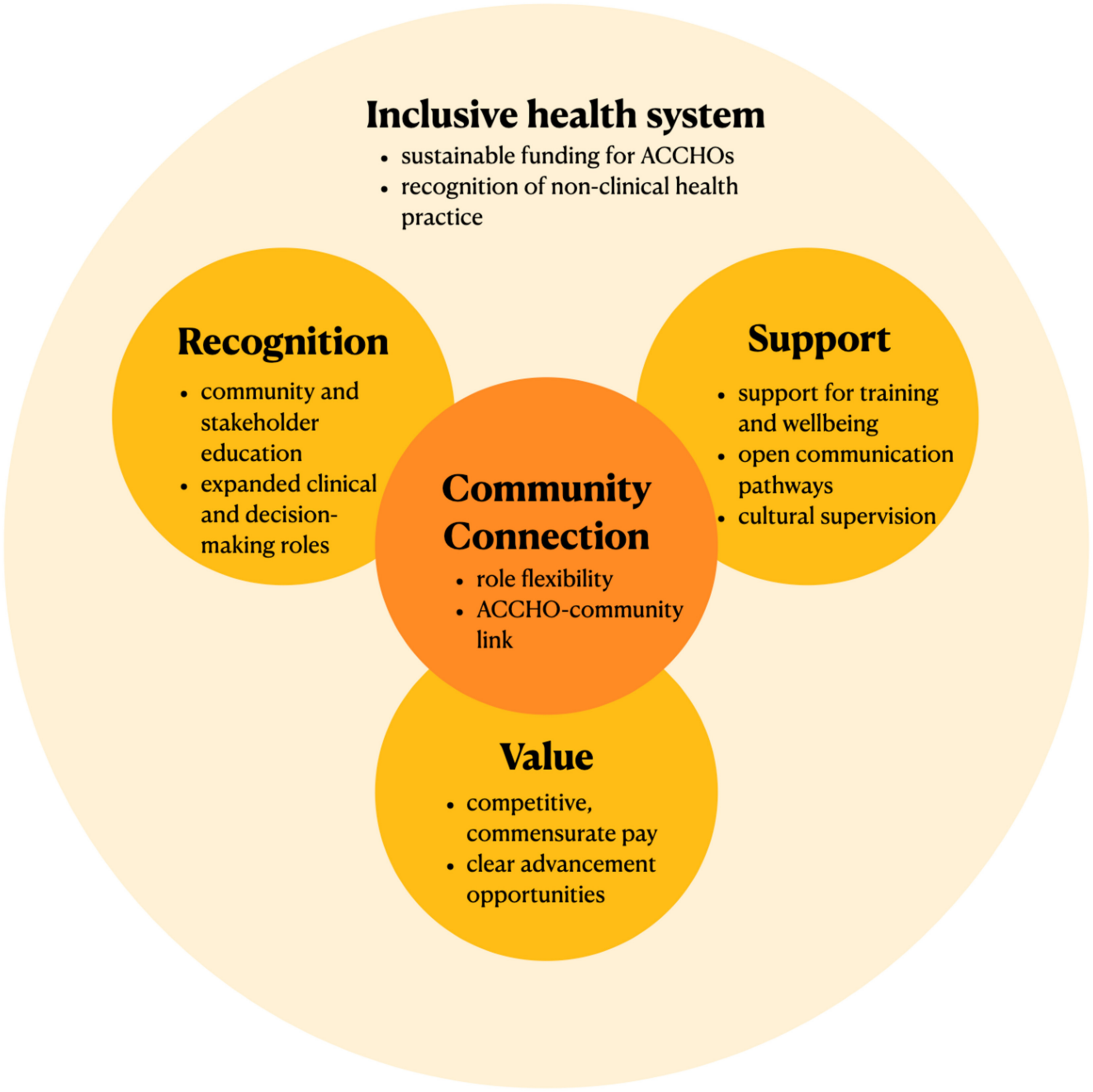
Actions to ensure AHW sustainability. This figure summarizes changes suggested by our yarns to potentially improve AHW sustainability within each of the five categories of change. AHW, Aboriginal Health Worker; ACCHO, Aboriginal Community Controlled Health Organisation.

These actions are summarized in Figure 6.

### 4.2. Implications of the five categories of change

The centrality of community connection to AHW practice is emphasized in existing literature on AHWs (8, 25, 44–46). In a mainstream health system, most healthcare positions are defined solely by what they do, or their role “responsibilities”. However, following yarning sessions with AHWs across Queensland, Topp et al. identified that beyond any role “responsibility”, community-centeredness forms a defining role “orientation” for AHWs.

Peiris et al. explain this orientation using the concept of *kanyini—*a term used by language groups across Central Australia(46). *Kanyini* is derived from an expression describing how a small child is held in one's arm against the breast (*kanyirnu yampungka*) (47). It is “the principle and primacy of caring for others–an obligation to nurture, protect and care for other people, family, and country” (46), and forms one of the four foundations of Aboriginal life, along with *Tjukurpa* (Law, Dreaming), *Walytja* (Family), and *Ngurra* (Land, Country) (47). *Kanyini*, with its notion of “holding”, is the foundation that motivates both AHW practice and the care provided to First Nations communities by ACCHOs. Any policy action involving AHWs must acknowledge and prioritize this foundation. The yarns suggested that community orientation could be supported through a role that is adequately flexible to adapt to the needs of the community. This has been echoed by Harris and Robinson following a study of AHWs in a mental health program in the Northern Territory (29). However, biomedical health systems such as Australia's are built on workforce plans which encourage specialization and focus on role responsibilities to create an “optimum skill mix” (9, 48). It is difficult to account for a highly flexible position, let alone its role orientation, in such a system. Indeed, Harris & Robinson found that the AHW role was only able to be “informally” incorporated into existing clinic structures, with non-pharmacological elements of care excluded in planning (29).

This aforementioned inability to fully account for AHWs within the NSW health system is at the center of their sustainability issues. For instance, our second category of change: the need for AHWs to gain recognition for their work, has already been highlighted by existing studies (49–53). An international review exploring First Nations health worker retention identified that recognition encompassed firstly, being entrusted to perform meaningful tasks, and secondly, feeling “seen” for having done so (52). Participants in our study suggested that expanding the AHW scope of practice to entrust more clinical and decision-making responsibilities will help improve recognition. Indeed, this will enable the recognition of AHW expertise by entrusting tasks that are considered “meaningful” within a biomedical model of health. However, it will still fail to gain recognition for the non-clinical expertise of AHWs–expertise which is most important in fulfilling their community orientation, but considered less “meaningful” within mainstream healthcare. The education of professionals, policymakers, and community members regarding the importance of the work of AHWs, as suggested by this study and others (7, 15, 26, 27), may help shift this paradigm. However, such change will require long-term, system-wide effort (15, 54).

The failure to recognize the holistic work of AHWs subsequently impedes the demonstration of value for their work. Each yarn in this study highlighted the importance of commensurate, competitive pay in attracting and retaining AHWs. This has been echoed in many other surveys of AHWs and stakeholders (50, 52, 55, 56). Presently, the AHW salary is fixed by a Commonwealth award (57). It is amongst the lowest of all staff salaries in the NSW health system (57, 58). Our yarns highlighted that ACCHOs may act to demonstrate value for their AHWs by negotiating salaries above the award and providing incentives such as training opportunities. However, ACCHOs in NSW are only partially funded, forcing them to rely on indefinite grants and block funding to finance these opportunities (24). Consequently, even service-level demonstrations of value are vulnerable to funding changes and are typically impermanent (24).

Ultimately, it is clear that to facilitate community connection, recognition, and value for AHWs, the health system must shift to become more inclusive of non-mainstream approaches to health. This will require policy change to implement workforce plans, funding models, and governance structures that are inclusive of non-clinical work and which center Aboriginal Ways of Knowing, Being, and Doing (24, 41). Recent strategies published by state and federal health bodies in Australia emphasize the importance of the AHW role in achieving health parity for First Nations peoples (18, 57). However, they rarely articulate firm commitments to better support the work of AHWs and ACCHOs. Further, many are written without the input of First Nations peoples—a characteristic shown to consistently contribute to policy failure (46).

The 2020 “National Agreement on Closing the Gap” (59) is a notable exception to this trend. The strategy, which has been co-designed by Australian governments and Aboriginal and Torres Strait Islander peak bodies, mandates that federal and state governments establish a “Community-Controlled Health Sector Strengthening Plan” by the conclusion of 2021(59). It is hoped that these plans will implement numerous strengths-based changes to build a more inclusive system for AHWs and ACCHOs.

In the absence of such system-wide change, our study suggests that AHW sustainability can continue to be promoted at a service level by focussing on the final category for change: support. A supportive workplace is recognized as vital to the retention of First Nations health workers (8, 60). Cultural support has been identified to be particularly paramount (8). Our yarns highlighted that AHW safety can be promoted by implementing pathways which ensure regular access to cultural supervision, such as regular Aboriginal and Torres Strait Islander staff meetings. Further, participants in our study recognized that professional support for AHWs was impeded by a lack of open communication between AHWs and management. This has been echoed elsewhere (60, 61). Implementing a “partnership model” in which regular opportunities for communication, group problem-solving, and interaction between staff are prioritized has been shown to effectively address these concerns and improve care for First Nations communities (26, 28).

### 4.3. Strengths and limitations

Overall, our study provides a comprehensive framework of factors affecting AHW sustainability in NSW. This framework can be used to guide service and system-level action to improve the AHW program. Our study also presents a replicable method that can be used to co-design culturally safe AHW studies across Australia. The core strength of our study was its use of Indigenous methodologies that centered on First Nations voices and knowledges. Additionally, despite the competing demands of the COVID-19 vaccination rollout, our study received a high response rate. All three sites willingly participated in the study, and 13 of the 21 employed AHWs participated in the yarning. The final sample of AHWs that participated in the yarn was representative of the diverse roles and backgrounds of staff across the sites, which aided the validity of our results.

Due to COVID-19 travel restrictions, the research team was unable to travel to the ACCHO sites to facilitate in-person yarns. This impeded “social yarning”, particularly in the initial yarns at each site. Bessarab and Ng'andu identified that social yarning is vital to building trust, accountability, and group rapport (36), and these links were delayed in our online yarns. Future online studies may account for this by dedicating extra time in initial sessions specifically for social yarning.

One limitation of our study was a small sample size. We collaborated with three regional centers. Given the highly variable nature of AHW practice and the distribution of ACCHOs across remote, regional, and metropolitan areas, these results are not generalisable to the entire AHW workforce in NSW. Co-designing a similar study with more ACCHOs across NSW will help identify specific actions to improve the AHW program and will help inform a more nuanced understanding of the systemic issues facing the NSW AHW program.

### 4.4. Conclusions

Yarns with AHWs and their supervisors working in PHC in NSW revealed five categories of change that are required to ensure AHW sustainability: community connection, recognition, value, support, and an inclusive health system. There are practical actions in each of these categories that can support AHW retention, such as implementing pathways for regular communication. However, it is evident that a system-wide paradigm shift in healthcare is necessary to truly ensure sustainability. The holistic work of AHWs and ACCHOs must be recognized through workforce plans, funding models and governance structures that incorporate Aboriginal Ways of Knowing, Being, and Doing. Co-designing similar studies with more ACCHOs across NSW can help inform this change.

## Data availability statement

The raw data supporting the conclusions of this article will be made available by the authors, without undue reservation.

## Ethics statement

The studies involving human participants were reviewed and approved by UNSW Human Research Ethics Committee (reference: HC210247) Aboriginal Health and Medical Research Council Human Research Ethics Committee (reference: 1800/21). The patients/participants provided their written informed consent to participate in this study.

## Author contributions

RJe conducted an initial literature review, coded the interview transcripts for analysis, and synthesized findings to draft this manuscript. RJo developed the original idea for the study in conjunction with BP and JC. JC established contact with potential Aboriginal health services across NSW to co-design the study, co-ordinated an Aboriginal Reference Group, and reviewed the study design and methods to ensure cultural safety. RJo created the yarning guide used in this study and provided a major contribution to the writing and analysis process. BP led the design of the thematic analysis process and also provided a major contribution to the analysis and writing of this study. All authors read and approved the final manuscript.
