# Supplementary material for: “We're on the ground, we know what needs to be done”: Exploring the role of Aboriginal Health Workers in primary health care
Source: Front Public Health. 2023 Jan 19;10:1010301. doi: 10.3389/fpubh.2022.1010301 (PMC9893014; doi:10.3389/fpubh.2022.1010301)
Supplement: Supplementary file 1 [file Data_Sheet_1.DOCX]

# Yarn Guide

*This supplementary file details the interview guide used by the research team to frame the yarns.*

***The aim of the meeting***

This yarn will be conducted to understand the current role and functioning of AHWs in delivering primary health care.

1. ***Role and recruitment***

How were you recruited to be an AHW?

Tell us about your current role? What are your responsibilities?

Can you tell us about your experience in providing primary health care?

How do you follow-up on your patients? And the rest of the community?

What do you find challenging in your role?

1. ***Training***

Tell us about the training that you received? Have you been evaluated as part of the training?

Did you feel you had adequate training for your role?

Do you usually receive any additional training (refresher/ongoing training) to help you in your role?

1. ***Accreditation***

Do you receive registration with professional bodies (e.g., AHPRA) for the training received? Could you tell us more about it?

1. ***Equipment and supplies***

Do you have the supplies and equipment you need to provide the services you are expected to deliver?

1. ***Supervision***

Could you tell us about the supervision you receive?

Who is your supervisor?

How often does your supervisor visit you or you visit he/she?

Do they usually accompany you in field visits or you meet them at the health centre?

Do you receive any feedback regarding your work from the community?

1. ***Incentives***

Is the remuneration you receive commensurate to the role and responsibilities?

Are there any non-financial incentives for your role?

1. ***Community involvement***

How does the community support the CHW program?

Do you feel that our role meets the expectations of the community?

Does the community provide any support to you in terms of feedback, support (financial/gifts in kind), formal recognition/appreciation?

1. ***Opportunity for advancement***

Are there opportunities for you to advance your career?

1. ***Data***

Do you collect any data as part of your job?

How does that data get back to the community and how is it used for quality improvement?

1. ***Linkage to the health system***

Do you refer patients to a hospital, allied health professionals, specialists, or other services?

Could you let us know the process involved?

Do you receive any feedback from the facility for patients that you have referred?

***End of session questions***

What are your biggest day-to-day challenges in doing your job?

What changes are needed to help you do your job better?
